# A Bone-Thickness Map as a Guide for Bone-Anchored Port Implantation Surgery in the Temporal Bone

**DOI:** 10.3390/ma6115291

**Published:** 2013-11-19

**Authors:** Jérémie Guignard, Andreas Arnold, Christian Weisstanner, Marco Caversaccio, Christof Stieger

**Affiliations:** 1 ARTORG Center for Biomedical Engineering, Artificial Hearing Research, University of Bern, Bern 3008, Switzerland; E-Mails: jeremie.guignard@artorg.unibe.ch (J.G.); andreas.arnold@insel.ch (A.A.); christof.stieger@usb.ch (C.S.); 2 Graduate School for Cellular and Biomedical Sciences, University of Bern, Bern 3012, Switzerland; 3 Department of ENT, Head and Neck Surgery, Inselspital, University Hospital of Bern, Bern 3008, Switzerland; 4 Department of Neuroradiology, Inselspital, University Hospital of Bern, Bern 3008, Switzerland; E-Mail: christian.weisstanner@insel.ch; 5 Department of ORL, University Hospital Basel, HNO Klinik, Hebelstr. 10, Basel 4031, Switzerland

**Keywords:** temporal bone, bone-anchored port, surgical guide, computer-assisted surgery, bonebridge

## Abstract

The bone-anchored port (BAP) is an investigational implant, which is intended to be fixed on the temporal bone and provide vascular access. There are a number of implants taking advantage of the stability and available room in the temporal bone. These devices range from implantable hearing aids to percutaneous ports. During temporal bone surgery, injuring critical anatomical structures must be avoided. Several methods for computer-assisted temporal bone surgery are reported, which typically add an additional procedure for the patient. We propose a surgical guide in the form of a bone-thickness map displaying anatomical landmarks that can be used for planning of the surgery, and for the intra-operative decision of the implant’s location. The retro-auricular region of the temporal and parietal bone was marked on cone-beam computed tomography scans and tridimensional surfaces displaying the bone thickness were created from this space. We compared this method using a thickness map (*n* = 10) with conventional surgery without assistance (*n* = 5) in isolated human anatomical whole head specimens. The use of the thickness map reduced the rate of *Dura Mater* exposition from 100% to 20% and suppressed sigmoid sinus exposures. The study shows that a bone-thickness map can be used as a low-complexity method to improve patient’s safety during BAP surgery in the temporal bone.

## 1. Introduction

There are several biomedical implants that use the temporal and parietal bones as implantation sites. The most common are implantable hearing aids. We can identify three types of temporal bone hearing implants: (1) Cochlear implants (CI) are systems, which electrically stimulate the auditory nerve through an electrode implanted in the cochlea. CIs typically need a bedding in the temporal/parietal region to host an electronic compartment and a magnetic coil [1]. In addition, the state of the art approach for CI implantation is to expose the round window in order to insert the electrode (facial recess approach); (2) Middle ear implants usually consist of a mechanical actuator implanted in a cavity burred in the mastoid or directly in the middle ear cavity. Such implants stimulate the cochlear fluid either by coupling with the ossicular chain, or directly at the round window (RW) [2,3]. Such devices also necessitate a bedding to host a transcutaneous induction antenna, similar to CI implantation; (3) Bone conduction implants generate a vibration of the skull, which is then transmitted to the inner ear through a phenomenon called bone conduction. These implants are either a percutaneous abutment implanted in the retro-aural region to which the actuator can be attached, such as for the bone-anchored hearing aid (Baha^®^), an active actuator implanted in the mastoid process with transcutaneous signal delivery, as for the Bonebridge^®^ [4], or a passive transcutaneous device, such as the Sophono Alpha1℘ [5].

There are other possible types of implants located in the temporal bone, such as a power connector to supply a heart-assisting pump [6] or an implantable inner ear drug delivery system [7,8]. 

**Figure 1:**
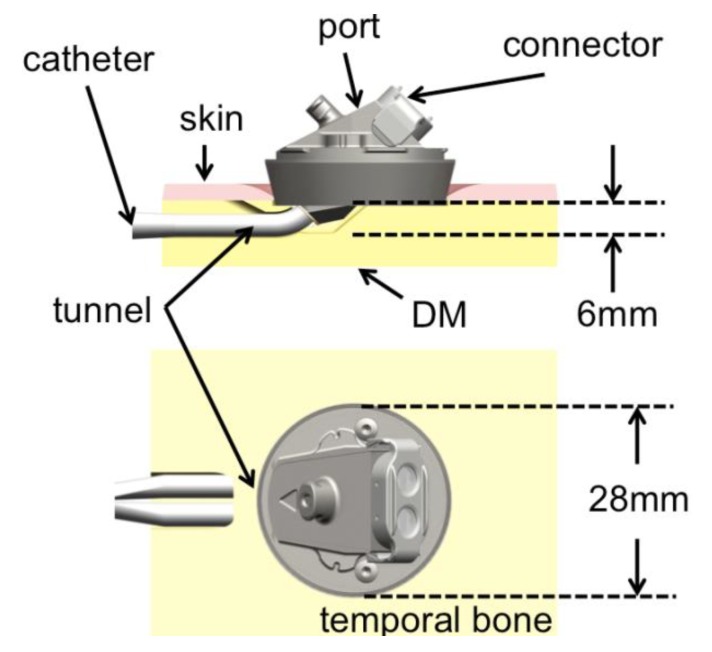
Schema of the implanted dialysis port, longitudinal section and view from above. A small tunnel is drilled in the bone to stabilize the skin surrounding the port. DM: *Dura Mater*.

In our institution, we investigate on the development of a bone-anchored vascular access port (BAP) with the temporal bone as an implantation site [9]. The goal is to take advantage of the long experience of stable percutaneous implants at the temporal region to provide a permanent vascular access with a lower complication rate. The BAP, currently under pre-clinical investigation, provides a percutaneous interface between a major blood vessel via a central venous catheter and an external connector (see Figure 1). The main application of the BAP is hemodialysis, although other therapeutic applications are possible [9].

During implantation surgery of all the devices listed above, injury to the *Dura Mater* (DM) or other vital anatomical structures hosted in the temporal bone, such as the sigmoid sinus (SS) and the facial nerve (FN) must be avoided to insure patient’s safety [10]. Surgeons rely on their anatomical knowledge and expose landmarks to safely drill the necessary cavities without injuring the above-mentioned vital structures. 

In the case of the BAP, a tunnel through which the catheter will be inserted has to be drilled (Figure 1). The tunnel allows the skin surrounding the implant to be in direct contact with bone, a situation that is believed to be favorable over a skin-catheter contact. Skin to bone contact has been observed to be mechanically and biologically stable [6]. In the process of creating this tunnel, the surgeon has to drill parallel to the cortical bone in a covered spot, which is normally not the case during standard mastoidectomy. Moreover, the thickness of the bone available in the temporal region is limited, and changes from patient to patient. Therefore, a method to display the bone thickness at a given location on the temporal-parietal region was developed to support the decision of the suitable implantation site. 

The use of computer planning for implant surgeries is gaining interest with the increasing availability of high-resolution computer tomography. The virtual fitting of implants in a computer model of the mastoid and middle ear cavity reconstructed from CT scans has been reported [7,11,12]. Other approaches to assist surgeons in the decision process of implant location are reported. They include navigated surgery, where an optical or electromagnetic system computes the relative position between the patient and a surgical tool using two sets of markers [13], and template-based surgery, where the position of the planned implantation site is constrained mechanically [14]. Some studies suggest the possibility of automatizing the entire planning and implantation through robotic surgery [15,16]. All methods listed above can achieve high accuracy but require some sort of reference system to be placed on the patient. For navigated surgery, a set of landmarks has to be defined in order to align planning data with the patient. These landmarks can be anatomical or artificial, where the latter has to be directly attached to the patient body, either glued on the skin, or attached to a dental splint. For high precision robotic surgery, artificial landmarks must even be fixed into the bone with screws, worn during a CT scan and kept until surgery, which adds a procedure and discomfort for the patient and also involves higher costs.

We propose a passive surgical guide in the form of a bone-thickness map based on a regular CT scan that can be presented to the surgeon during the implantation procedure and that relies exclusively on anatomical landmarks. The aim is achieve the necessary accuracy for the safe surgery for implants in the temporal bone, and thus reduce the risk for the patient, while keeping the complexity low. We evaluated the bone-thickness map on cadaver head specimens during BAP test implantation surgeries.

## 2. Method

### 2.1. Specimens and Image Acquisition

We used a total of 15 ears from 11 cadaver heads embalmed according to Thiel [17] for this study (Table 1). The surgical procedure of the BAP was performed without thickness-map in 5 heads. In the other 10 ears, the 3D scans were obtained before surgery, using a cone-beam computer tomography (CBCT) X-ray unit (Promax^®^ 3D Max, Planmeca Oy, Helsinki, Finland). The specimens were placed in a plastic container at the center of the revolving scanning arm. We used a lower skull scan protocol with the following parameters: 96 kV, 12 mA, 230 mm FOV, 108 mAs and a slice thickness of 0.4 mm. The focal spot was 0.6 mm × 0.6 mm according to the manufacturer’s documentation. The reconstruction of the images was achieved with the Romexis^®^ software (Planmeca Oy, Helsinki, Finland). The resulting image stacks had an isotropic voxel size of 0.4 mm and were saved in DICOM format. All image stacks were inspected and no pathologies or malformations of the temporal bone were found.

### 2.2. Bone-Thickness Map Generation

The bony structure of the temporal-parietal region was labeled on the scan images (a process called segmentation) using Amira^®^ 5 (VSG, Burlington, MA, USA). CBCT intensity is related to the density of the scanned structures, which allows for density-selective labeling. The labeling was achieved using a conditional threshold and semi-automatic edge-detection tools. The temporal/parietal region contains zones of lower density, such as trabecular bone, mastoid air cells and middle ear cavities. The mastoid cortex and air-cells were marked as inside in the labeling process as middle ear cavities were marked as outside. A 3D surface made of approximately 100,000 small triangles was generated from the labeled images using Amira’s built-in surface generation script. This surface was separated into an external and an internal surface, such that the external surface represents the surface of the skull and the internal one, the surface of the DM, the SS, and the FN. For each point of the external surface, the Euclidian distance to the closest point of the internal surface was computed using the built in surface-distance script of Amira. 

Formally, the inner and outer surfaces can be described as discrete collections of triangles in space. The distance map between surface A and B is a four-dimensional vector field containing points with the same coordinates as the elements of A, and a value representing the distance to the closest point in B. 

**Figure 2:**
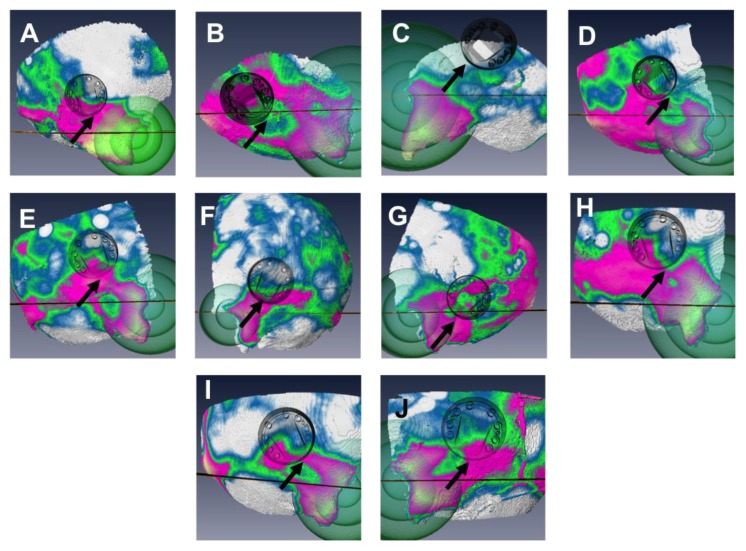
The distance map and actual position of the port after guided surgery in the chronological order (**A**–**J**). The map shows 4 zones (white: <5 mm; blue: (5–6) mm; green: (6–7) mm; pink/yellow >7 mm). The reference information such as isodistances to the Henle’s spine (green spheres) and the prolongation of the zygoma line (black line) are visible. The black arrow indicates the location of point of deepest necessary drilling.

By assigning a color value to the distance, the distance scalar field could be drawn in perspective. The color map was set so that it showed the portions of the bone with a thickness above 5, 6, and 7 mm in different colors (Figure 2). To allow the surgeon to transfer a position on the map to the patient and *vice versa*, a reference system based on two anatomical landmarks was added to the map. The landmarks chosen were the Henle’s spine (HS) and the line of the zygoma (ZL). The HS is a small bony prominence anterior to the supramastoid pit at the posterosuperior margin of the bony external auditory canal. The HS is easily accessible via the retro-auricular incision necessary for the implantation. The ZL can be found by palpation and prolonged, either mentally or with a surgical ruler. The isodistances to the HS were shown using concentric transparent spheres and the prolongation of the zygoma with a black line (Figure 2). The process of generating the thickness map took between 1.5 and 2 h per case, depending on the difficulty of the segmentation. A detailed tutorial to reproduce the method used to create the map can be found online [18]. 

### 2.3. Surgery

The cadaver specimens were stabilized on a ring-shaped soft cushion. A retro-auricular incision was performed and the skin was elevated to uncover the temporal-parietal region. The bone-thickness map was presented to the surgeon as a guide to choose the ideal implant location. The surgeon had to select the position in order to have the point with the deepest drilling where the bone was thicker than 6 mm (Figure 2). The cavity that needed to be drilled was 6 mm deep at most, and covered an area of roughly 10 mm × 5 mm. The other criteria were that the port could not be in contact with the auricle, and that the bone should be as flat as possible at this point to allow a stable fixation of the port. A surgical ruler was used to transfer the position relative to the HS and the ZL from the map to the specimen. The prototype was implanted using standard otologic surgical equipment and a custom made drilling caliber.

The procedure was done by a total of three different experienced ENT surgeons, all of which were familiar with the novel implant (Table 1). The reason for requesting several surgeons is to prevent the evaluation of the method to be biased towards one, well-trained surgeon. For 5 ears, the surgery was performed without map guidance. 

### 2.4. Postoperative Analysis

To assess the postoperative status of the critical structures, the integrity of the bone layer was inspected visually and with a surgical probe as well as on postoperative CBCT-scan images in all 15 cases. To compute the actual position of the port and compare it with the thickness map, the pre- and postoperative image stacks were rigidly registered (*i.e*., aligned with only translations and rotations) using a rigid landmark warp. Landmarks were chosen among the ones close to the ear and not affected by the surgery: the left and right HS, and the tip of the left and right mastoid portion of the temporal bone. The position of the center of the port relatively to the HS and the ZL was recorded for all experiments using the built-in 3D distance measurement tool of Amira. 

To assess the accuracy of the distance measurement, one specimen with a set of four radiological markers attached to surgical screws was scanned with the same CBCT protocol. A 3D model of the specimen and the screws was reconstructed in Amira as described above. The relative distances between the screws heads measured on the virtual model were compared to the corresponding distances measured on the specimen with a mechanical caliber. The error was less than 0.5 mm.

## 3. Results

The integrity of the critical structures after surgery is reported in Table 1. The DM was systematically exposed in unguided surgeries. In the following 10 guided surgeries the exposure of the DM occurred in two cases and was therefore significantly lower (Mann Whithney test, *p* < 0.01). The rate of intact, (not exposed, not skelotonized) SS increased from 40% for the unguided surgeries to 100% in the guided surgeries. The facial nerve was left intact in all experiments. The uncovering of the DM in experiment 14 was due to excessive drilling under the port. 

The individual preoperative bone-thickness maps as well as the actual position of the port, measured post-operatively in the 10 guided surgeries is shown in Figure 2. The bone was systematically thicker in the fronto-caudal portion of the temporal bone, which was therefore a preferred site of implantation. It can be seen that the port was placed at a location where the point of deepest drilling (highlighted with an arrow) is located in the 6–7 mm region for most cases. A notable exception is experiment number 8 (Figure 2C), which led to uncovering of the DM (Table 1). The maps show a significant variation in overall bone thickness and thickness patterns between individuals.

**Figure 3:**
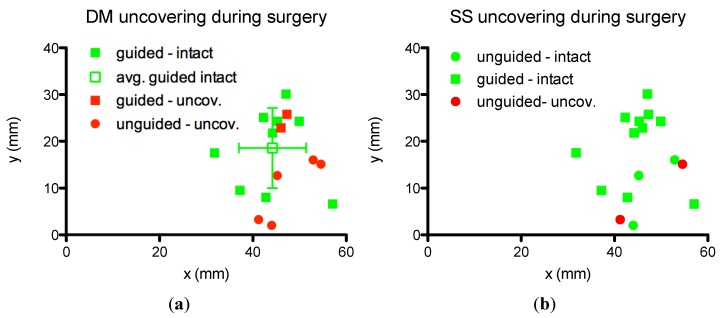
Position of the port relatively to the Henle spine (point 0,0) and the zygoma line (*x*-axis). Green: intact structure; red: surgeries where (**a**) the DM; or (**b**) SS was uncovered; dots: unguided surgeries; squares: guided surgeries. The average and standard deviation of the position by guided surgeries leaving all structures intact.

The position of the port’s center relatively to the HS and the ZL is reported in Figure 3. The position of the port when the DM and SS were uncovered is indicated in red. The port was located, on average, at 45.55 ± 6.3 mm posterior from the HS and 16.55 ± 8.7 mm cranial from the prolongation of the ZL. No clear difference in the position can be seen between surgeries where the DM was uncovered and those were it was left intact. Guided surgeries where done more cranial on average than unguided surgeries although no net separation can be seen.

**Table 1:** List of the experiments. DM: Dura Mater; SS: sigmoid sinus; FN: facial nerve; U: uncovered; S: skeletonized; I: intact. Letters with the experiment number indicate the respective image on Figure 2.

Experiment	Specimen	Side	Age	Sex	Surgeon	DM	SS	FN
Unguided surgeries:								
1	K01	R	88	M	1	U	I	I
2	K02	R	98	F	1	U	U	I
3	K03	R	79	F	2	U	U	I
4	K04	R	78	F	3	U	S	I
5	K05	R	50	F	1	U	I	I
Summary unguided	–	100% R	average: 78.6 ± 17.9	20% M 80% F	–	100% U	40% I 20% S 40% U	100% I
Guided surgeries:								
6-A	K06	R	84	M	1	I	I	I
7-B	K07	R	91	F	1	I	I	I
8-C	K06	L	84	M	2	U	I	I
9-D	K08	R	83	M	2	I	I	I
10-E	K09	R	78	F	1	S	I	I
11-F	K09	L	78	F	2	S	I	I
12-G	K04	L	78	F	2	I	I	I
13-H	K10	R	83	F	1	S	I	I
14-I	K11	R	86	M	2	U	I	I
15-J	K11	L	86	M	2	S	I	I
Summary guided	–	60% R 40% L	average: 83.3 ± 4.5	42.8% M 57.2% F	–	40% I 40% S 20% U	100% I	100% I

## 4. Discussion

In all cases, the implantation of the port prototype was safe in regard of the FN, as the implantation procedure is carried out more superficially (max. depth 6 mm) to the normal anatomical course of the mastoid FN portion. However, during unguided surgeries, there was a systematical exposure of the DM, which could be significantly reduced with the thickness map. SS exposure was not observed in the guided group. In one case of the guided group where the DM was exposed, the post-surgery CT images showed that it was due to excessive drilling (the surgeon drilled deeper than the 3–4 mm necessary for the bedding). The other case of uncovered DM was due to a misplacement of the port (Figure 2C). The misplacement of the port in that case was most likely a consequence of inaccurate transfer of the position on the map to the position on the head specimen. The zygoma line must be mentally prolonged by the surgeon and is, thus, prone to uncertainty. The statistical test must be interpreted with caution, because of the relatively small sample size.

It was not possible to blind the experiments, as the surgeons automatically knew whether the map was used or not in a given experiment. To reduce the potential bias due to this situation, we asked the surgeons to conduct all surgeries without guidance first, and then introduced the map for consecutive surgeries. The reason for requesting several surgeons was to demonstrate that the implantation is not limited to a single specially trained surgeon. The positive effect of the bone-thickness map on safer implantation was instantly notable, suggesting that the map may be used to its full potential the first times the surgeon uses it. Generating the map, however, necessitate experience in segmentation of the temporal/parietal region and a comprehensive knowledge of the ear’s anatomy.

It was not possible to see a separation in the position of the port between surgeries leaving the DM intact and those were it was uncovered (Figure 3). It is the guided/unguided criterion that has an influence on the rate of critical structure uncovering, not the position of the port relatively to the ear canal. If a position criterion could be found (such as the one used clinically for bone-anchored hearing implants) it would have been more practical than the map. As it can be seen in Figure 2 the variations in thickness and thickness pattern between individuals do not allow the definition of a patient-independent preferred implantation site. The inter-individual variation of the available room in the mastoid has been already discussed [7]. 

It is known that segmentation of ear images on computer tomography images can lead to volume and thickness error. The main reasons reported are movement artifacts and partial volume effect [19]. Movement artifacts are irrelevant when considering cadaveric specimens. We estimated the segmentation error due to partial volume effect to be minimal in this region of the skull because the slice thickness (0.4 mm) is small relative to the in-plane changes in shape between consecutive slices. Moreover, as discussed in the literature, segmentation on CBCT images tend to slightly underestimate structures’ volume [20]. Thus, we estimated errors in thickness to be negligible and more likely to be underestimations of the thickness, which is less critical than overestimations. 

The choice of colors for the map was subject to optimization. At first, red and green were used to indicate regions where the thickness was insufficient and sufficient respectively. Problems arose with this choice, as some people have difficulties distinguishing the two. The colors chosen allow to clearly distinguish the different zones, whether on a screen, or printed in colors or gray scale. In particular, to highlight regions where the implantation was not indicated, white was chosen as it is easily differentiated from the others. 

During preliminary experiments (data not shown), we implemented one additional element for the reference system: the skull sutures. The sutures, especially the temporal-parietal and parietal-occipital sutures, can be seen on the CBCT images and can be segmented as a separate structure and displayed on top of the map. While the sutures allow a slightly more accurate transfer of a position in the virtual model to the patient, segmentation requires a significant additional workload. We chose not to include this additional landmark because the additional value did not counterbalance the additional workload. 

In addition to a surgical guide, the bone-thickness map can be used as an inclusion/exclusion criterion for implant surgery, as it shows if the bone is sufficiently thick at the region typically chosen for the implantation, e.g., patients with bone thickness lower than 6 mm in the temporal bone region should be excluded from BAP implantation.

The current method requires approximately 2 h for the generation of the surgical guide from the CT images. In comparison with a standard hearing implant surgery it is relatively long. The most tedious part is the segmentation, which requires comprehensive anatomical knowledge and manual labeling. The specificity of the temporal/parietal region, with imbricated high and low density structures makes it impossible to segment by simple threshold filters or region growing algorithms. In the future, if automatic segmentation can be achieved (for example using statistical shape models [21]) the overall procedure time could be drastically reduced.

The presented bone thickness map may be a low-complexity and efficient method to reduce the risk of injuries during implantation surgery in the temporal bone.

## 5. Conclusions and Outlook

We have shown that a bone-thickness map can be used to plan the position of an implant in the temporal bone to increase the patient’s safety, while keeping a low complexity of the additional procedure. The use of the map reduced DM exposure and suppressed SS exposures. The advantage of this method is that it gives additional information to the surgeon without constraining the implantation site (a method also known as a passive surgical guide). It allows the surgeon to take other aspects into account, such as the relative distance to the auricle and the quality and evenness of the bone under the implant. It also does not require a reference system to be placed on the patient or additional equipment to be taken into the operating theater.

The following step is the clinical study. While this cadaver study gives the proof of concept for the surgical procedure, its success will depend on the outcome of its use on patients.

Recently, the bone thickness map was introduced in our institution, in the workflow of another device’s implantation. The Bonebridge^®^ is a new type of bone-conduction implant, which necessitates a cavity measuring about 9 mm in depth and 8 mm in diameter to be drilled in the mastoid. As can be seen in Figure 2, this cavity is not easily burred away in all patients. Thus far, five (*in vivo*) surgeries were conducted with evaluation and guidance with a bone-thickness map, which shows that its possible application goes beyond the bone-anchored port it was initially developed for. The clinical validation of this new procedure is ongoing and will be the subject of another publication.
